# Mitigation of Cadmium and Copper Stress in Lettuce: The Role of Biochar on Metal Uptake, Oxidative Stress, and Yield

**DOI:** 10.3390/plants14152255

**Published:** 2025-07-22

**Authors:** Riccardo Fedeli, Zhanna Zhatkanbayeva, Rachele Marcelli, Yerlan Zhatkanbayev, Sara Desideri, Stefano Loppi

**Affiliations:** 1 BioAgry Lab, Department of Life Sciences, University of Siena, 53100 Siena, Italy; loppi@unisi.it; 2 Department of Life Sciences, University of Siena, 53100 Siena, Italy; r.marcelli1@student.unisi.it (R.M.); s.desideri2@student.unisi.it (S.D.); 3 Laboratory of Engineering Profile, L.N. Gumilyov Eurasian National University, Satpaev Str., 5, Astana 010008, Kazakhstan; zhanna01011973@mail.ru; 4 Department of Chemistry and Ecology, K. Kulazhanov Kazakh University of Technology and Business, Astana 010000, Kazakhstan; erlan.ntp@mail.ru; 5 NBFC, National Biodiversity Future Center, 90121 Palermo, Italy

**Keywords:** bioavailability, crop plants, heavy metal remediation, metal immobilization, sustainable agriculture

## Abstract

Biochar has emerged as a promising soil amendment for mitigating heavy metal contamination in agricultural systems. This study investigates the effects of biochar on cadmium (Cd) and copper (Cu) uptake, plant growth, oxidative stress, and physiological responses in lettuce (*Lactuca sativa* L.) plants exposed to different metal concentrations. Results indicate that biochar significantly influenced Cd bioavailability, reducing its accumulation in plant tissues by up to 31.9% and alleviating oxidative stress, with malondialdehyde and proline levels decreasing by up to 51.0% and 60.2%, particularly at higher application rates (5%). Cd-exposed plants treated with biochar exhibited an improved fresh weight (+22.6%), lower malondialdehyde and proline levels, and enhanced the chlorophyll content (+14.9% to 24.1%) compared to untreated plants. The bioaccumulation factor for Cd decreased (up to 31.8%) while the immobilization index (II) increased, confirming the role of biochar in limiting Cd mobility in soil. In contrast, Cu uptake remained consistently low across all treatments, with a significant reduction observed only at higher contamination levels (up to −34.2%). Biochar contributed to Cu immobilization, reflected in increased II values, and enhanced the plant biomass and chlorophyll content under Cu exposure (+15.4% and up to +24.1%, respectively), suggesting a partial alleviation of Cu toxicity. These findings highlight biochar’s potential in heavy metal remediation, particularly for Cd, by reducing bioavailability and improving plant resilience. However, its role in Cu-contaminated soils is mainly through immobilization rather than uptake reduction.

## 1. Introduction

Soil contamination by cadmium (Cd) and copper (Cu) is of great environmental concern for agriculture [1,2]; these elements are among the most relevant contaminants due to their persistence in the environment, leading to long-term accumulation [3,4], toxicity to plants and microorganisms [5,6], and potential risks to human health [7,8].

Copper contamination is often associated with the extensive use of copper-based agrochemicals, such as fungicides and pesticides [5,9], and although Cu is an essential micronutrient for plants [10], excessive concentrations can lead to phytotoxicity [11], inhibiting plant growth and microbial activity in the soil [12]. Cadmium, on the other hand, is mainly introduced into soils through phosphate fertilizers [13], and, unlike Cu, it has no known biological function and is highly toxic even at low concentrations, posing severe risks to human health through food chain biomagnification [14]. Given these challenges, effective and sustainable strategies are urgently needed.

Biochar, a carbon-rich material produced through the pyrolysis of organic biomass under limited oxygen conditions [15], is gaining attention as a cost-effective and environmentally friendly alternative for mitigating toxic metal contamination in soils [16,17]. Biochar production transforms agricultural waste, wood residues, and other biomass sources into a stable form of carbon that can be applied to soils to improve their physicochemical properties [18]. Biochar is known for its high porosity, large surface area, and capacity to adsorb pollutants, making it an effective amendment for immobilizing toxic metals and reducing their bioavailability to plants [19,20]. Several mechanisms contribute to the ability of biochar to remediate contaminated soils. First, it can increase soil pH, thus reducing the metal solubility and enhancing their immobilization [21]. Second, it contains functional groups (i.e., carboxyl, hydroxyl, and phenolic groups) that can form complexes with metal ions, thereby reducing their mobility [22]. Third, it improves soil structure, water retention, and microbial activity, indirectly promoting plant health and resilience to metal toxicity [23,24,25].

Some studies have shown that the application of biochar significantly reduces the availability of Cd and Cu in contaminated soils, thereby mitigating their phytotoxic effects and preventing their accumulation in crops [26]. However, the effectiveness of biochar depends on several factors, including feedstock type, pyrolysis conditions, soil characteristics, and contamination levels [27]. Understanding these interactions is crucial for optimizing biochar applications in different environmental and agricultural contexts.

In Italy, the soil contamination of agricultural soils is regulated by law [28,29], which set well defined environmental quality standards, i.e., 200 mg kg^−1^ for Cu and 5 mg kg^−1^ for Cd, to prevent uptake by crop and subsequent entry into the food chain, with potential risks for human health. Industrial/commercial soils have higher permissible contamination levels: 600 mg kg^−1^ for Cu and 15 mg kg^−1^ for Cd [28]. Exceeding these limits requires intervention measures, ranging from containment and monitoring to active remediation strategies. The ability of biochar to immobilize toxic metals without disrupting soil ecosystems makes it a promising solution for compliance with the above environmental quality standards. Moreover, the Provisional Tolerable Weekly Intake (PTWI) established by the FAO/WHO is 7 µg kg^−1^ body weight for Cd and 3 mg/day for Cu (adult reference).

This study was guided by the hypothesis that a biochar addition to Cd- (5 mg kg^−1^ and 15 mg kg^−1^) and Cu-contaminated (200 mg kg^−1^ and 600 mg kg^−1^) soils can immobilize metals, thereby reducing their uptake by plants, alleviating oxidative stress, and improving lettuce growth and yield. To test this, the specific objectives are as follows: (i) to evaluate biochar’s effect on soil metal bioavailability; (ii) to quantify Cd and Cu accumulation in lettuce tissues; (iii) to assess oxidative stress responses; and (iv) to determine changes in biomass and yield under biochar amendment.

## 2. Results

The addition of biochar resulted in different responses to Cd and Cu exposure; all comparisons are with plants grown with B_0_.

**Figure 1:**
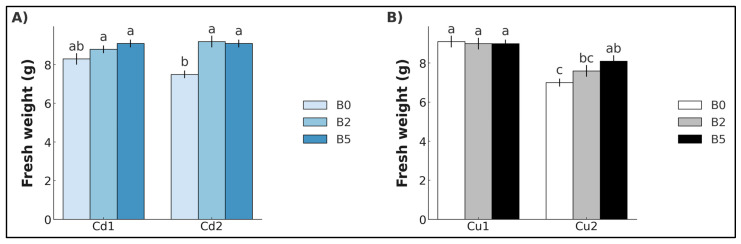
Fresh weight (mean ± SE, *n* = 10) of lettuce plants grown in soil contaminated with Cd (**A**) and Cu (**B**). Cd_1_ = Cd 5 mg kg^−1^; Cd_2_ = Cd 15 mg kg^−1^; Cu_1_ = Cu 200 mg kg^−1^; Cu_2_ = Cu 600 mg kg^−1^. B_0_ = biochar 0%; B_2_ = biochar 2%; B_5_ = biochar 5%. Different letters indicate statistically significant differences between the treatments (*p* < 0.05).

As far as fresh weight is concerned, no significant differences were observed in plants exposed to Cd_1_ concentrations, but a statistically significant increase was recorded in lettuce grown with B_2_ (+22.6%) and B_5_ (+21.2%) (Figure 1A). Similarly, no significant differences were found in plants exposed to Cu_1_ concentrations, whereas a statistically significant increase was observed in lettuce grown with B_5_ (+15.4%) (Figure 1B).

**Figure 2:**
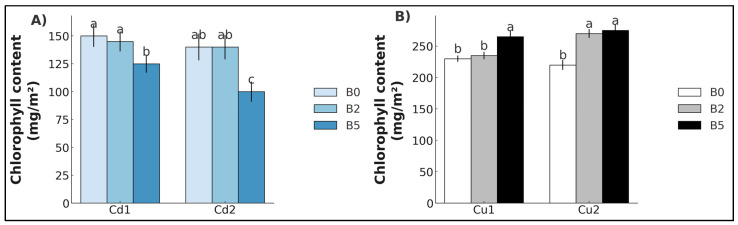
Chlorophyll content (mean ± SE, *n* = 10) of lettuce plants grown in soil contaminated with Cd (**A**) and Cu (**B**). Cd_1_ = Cd 5 mg kg^−1^; Cd_2_ = Cd 15 mg kg^−1^; Cu_1_ = Cu 200 mg kg^−1^; Cu_2_ = Cu 600 mg kg^−1^. B_0_ = biochar 0%; B_2_ = biochar 2%; B_5_ = biochar 5%. Different letters indicate statistically significant differences between the treatments (*p* < 0.05).

Regarding the chlorophyll content, B-grown plants exposed to Cd_1_ and Cd_2_ exhibited a statistically significant reduction at the B_5_ concentration (−15.4% and −29.6%, respectively) (Figure 2A). In contrast, an increase was detected in Cu_1_ under B_5_ (+14.9%) and in Cu_2_ for both B_2_ and B_5_ (+21.1% and 24.1%, respectively) (Figure 2B).

**Figure 3:**
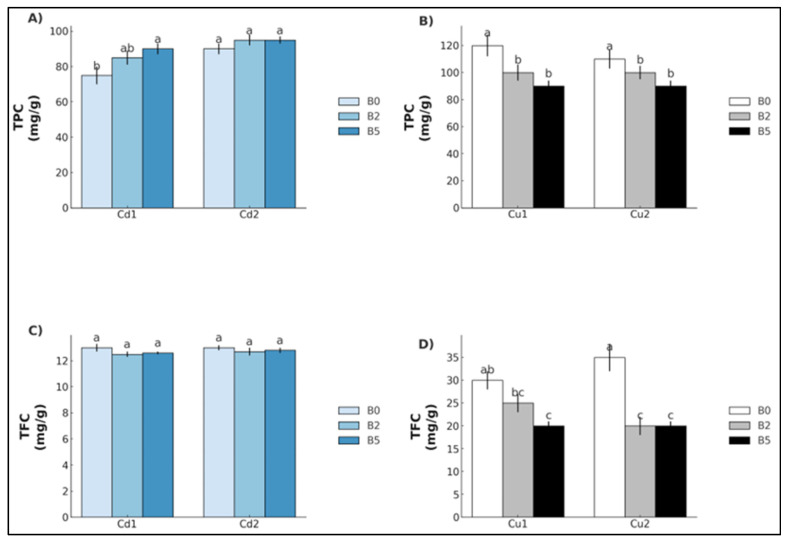
Total phenolic content (TPC) and total flavonoid content (TFC) (mean ± SE, *n* = 10) of lettuce plants grown in soil contaminated with Cd (**A**,**C**) and Cu (**B**,**D**). Cd_1_ = Cd 5 mg kg^−1^; Cd_2_ = Cd 15 mg kg^−1^; Cu_1_ = Cu 200 mg kg^−1^; Cu_2_ = Cu 600 mg kg^−1^. B_0_ = biochar 0%; B_2_ = biochar 2%; B_5_ = biochar 5%. Different letters indicate statistically significant differences between the treatments (*p* < 0.05).

In terms of the total phenolic content (TPC) and total flavonoid content (TFC), Cd exposure led to a statistically significant increase in TPC only in Cd_1_ plants grown with B_5_ (+32.3%), while no differences were found for Cd_2_ (Figure 3A). TFC showed no significant variations in either Cd_1_ or Cd_2_ (Figure 3C). Under Cu exposure, TPC significantly decreased in Cu_1_ and Cu_2_ for both B_2_ and B_5_ (Cu_1_: −23.64% and −30.74; Cu_2_: −21.7% and −23.5%) (Figure 3B). TFC also showed a statistically significant reduction in Cu_1_ under B_5_ (−35.6%) and in Cu_2_ for both B_2_ and B_5_ (−53.1% and −46.6%, respectively) (Figure 3D).

**Figure 4:**
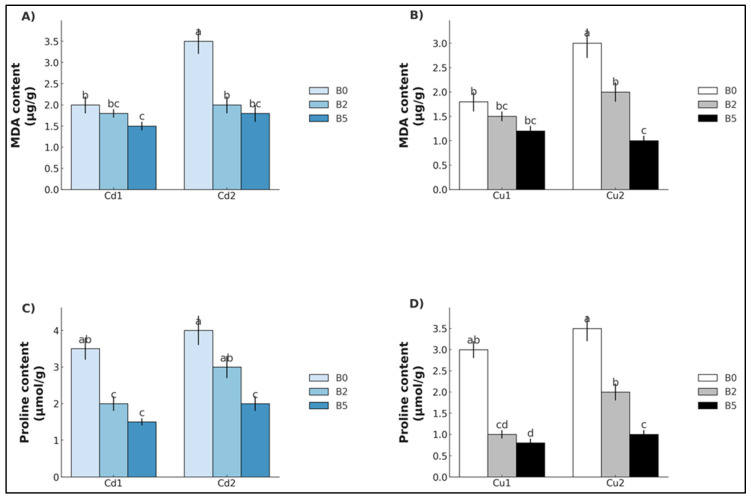
Malondialdehyde (MDA) and proline content (mean ± SE, *n* = 10) of lettuce plants grown in soil contaminated with Cd (**A**,**C**) and Cu (**B**,**D**). Cd_1_ = Cd 5 mg kg^−1^; Cd_2_ = Cd 15 mg kg^−1^; Cu_1_ = Cu 200 mg kg^−1^; Cu_2_ = Cu 600 mg kg^−1^. B_0_ = biochar 0%; B_2_ = biochar 2%; B_5_ = biochar 5%. Different letters indicate statistically significant differences between the treatments (*p* < 0.05).

For oxidative stress indicators, malondialdehyde (MDA) levels in Cd-exposed plants only showed significant differences in Cd_1_ for B_5_ (−27.9%), whereas a statistically significant reduction was observed in Cd_2_ for both B_2_ and B_5_ (−35.1% and −51.0%, respectively) (Figure 4A). For Cu-exposed plants, no significant differences emerged for Cu_1_, whereas a statistically significant reduction was observed in Cu_2_ for both B_2_ and B_5_ (−46.7% and -69.3%, respectively) (Figure 4C). Proline levels, on the other hand, were significantly reduced in both cases for Cd and Cu under B_2_ and B_5_ (Cd_1_: −56.4% and −49.6%; Cd_2_ −18.8% and −60.2%; Cu_1_: −65.4% and −71.9%; Cu_2_ −29.8% and −58.7%) (Figure 4B,D).

**Figure 5:**
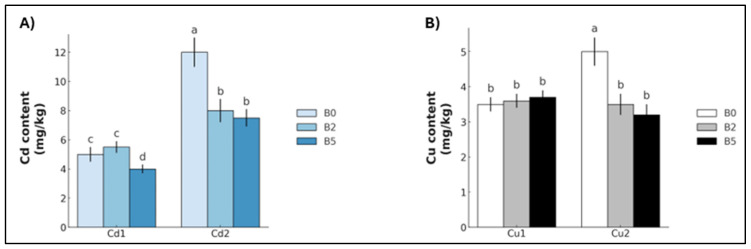
Cadmium (Cd) and copper (Cu) content (mean ± SE, *n* = 10) of lettuce plants grown in soil contaminated with Cd (**A**) and Cu (**B**), respectively. Cd_1_ = Cd 5 mg kg^−1^; Cd_2_ = Cd 15 mg kg^−1^; Cu_1_ = Cu 200 mg kg^−1^; Cu_2_ = Cu 600 mg kg^−1^. B_0_ = biochar 0%; B_2_ = biochar 2%; B_5_ = biochar 5%. Different letters indicate statistically significant differences between the treatments (*p* < 0.05).

Metal uptake also varied between Cd and Cu. Cd_1_ plants exhibited a statistically significant reduction in Cd accumulation only under B_5_ growth (−31.9%), whereas Cd_2_ plants showed a significant decrease for both B_2_ and B_5_ (−29.8% and −27.0%, respectively) (Figure 5A). In contrast, Cu uptake showed no significant differences at Cu_1_, but a statistically significant reduction was recorded in Cu_2_ for both B_2_ and B_5_ (−27.0% and −34.2%, respectively) (Figure 5B).

In terms of BAF, Cd_1_ showed a statistically significant reduction only under B_5_ (−31.8%), while Cd_2_ showed a significant decrease for both B_2_ and B_5_ (−28.9% and −25.7%, respectively). For Cu, no significant differences were observed in Cu_1_, whereas Cu_2_ exhibited a statistically significant reduction for both B_2_ and B_5_ (−37.5% and −37.6%, respectively) (Table 1).

Regarding the II, the immobilization effect increased with higher B concentrations. In terms of Cd_1_, low immobilization was observed for B_2_, but a stronger effect was detected for B_5_. For Cd_2_, both B_2_ and B_5_ led to significant immobilization. A similar trend was observed for Cu, where low immobilization was detected for Cu_1_, while Cu_2_ showed higher immobilization under both B_2_ and B_5_ treatments (Table 1).

## 3. Discussion

### 3.1. Cadmium

Cadmium is a highly toxic metal with no biological function in plants [14]. Its strong mobility in soil and high bioavailability lead to uncontrolled accumulation in plant tissues, causing severe physiological and biochemical stress [30]. Our study found that Cd exposure, particularly at 15 mg kg^−1^ (Cd_2_), resulted in significant oxidative damage, growth inhibition, and metabolic disturbance. However, biochar, especially at 5%, effectively mitigated Cd toxicity by reducing its bioavailability, improving plant physiological responses, and enhancing antioxidative defenses.

Cadmium toxicity severely reduces plant growth by impairing nutrient uptake, disturbing hormonal balance, and inducing oxidative stress [31]. Our results showed that Cd_2_-treated plants exhibited a significant reduction in fresh weight compared to Cd_1_, confirming the dose-dependent effect of Cd toxicity. However, B amendments, particularly at 5%, significantly increased fresh weight under both Cd_1_ and Cd_2_ conditions, suggesting improved growth. This aligns with findings by Houben et al. [32], who reported that B amendment reduces Cd bioavailability and improves plant biomass production in contaminated soils. Specifically, in spinach (*Spinacia oleracea* L.) plants, the application of 1.2% biochar to soil containing Cd (20 mg/kg) increased shoot fresh weight by 86.2% and root fresh weight by 96.2% [33]. Similarly, in lettuce plants, the application of 5% biochar to soil containing Cd (10 mg/kg) significantly increased fresh weight [34].

It is well known that Cd disrupts chlorophyll biosynthesis by inhibiting key enzymes such as δ-aminolevulinic acid dehydratase and protochlorophyllide reductase, leading to reduced photosynthetic efficiency [35]. Our results confirmed that Cd_2_ conditions significantly reduced the chlorophyll content in B_0_ plants, likely due to increased oxidative stress and metal interference with chlorophyll metabolism. However, B application, particularly B_5_, cannot restore the chlorophyll content, indicating that a higher concentration of Cd is truly toxic to the plants and may surpass the mitigation capacity of B. This suggests that while B can alleviate some Cd-induced stress, its effectiveness diminishes at elevated Cd levels, leading to persistent chlorophyll degradation.

Phenolic compounds and flavonoids play a vital role in plant defense mechanisms against heavy metal stress. Cd exposure significantly increased the total polyphenol content (TPC) and total flavonoid content (TFC) in plants, particularly under Cd_2_ conditions. This response is consistent with previous findings, where Cd toxicity induced oxidative stress, triggering an upregulation of antioxidant metabolites to neutralize reactive oxygen species (ROS) [36]. However, plants grown with biochar showed a more balanced antioxidant response, with moderate TPC and TFC levels, suggesting that biochar reduced the need for excessive antioxidant synthesis by alleviating Cd-induced stress. This aligns with previous research demonstrating the ability of biochar to regulate oxidative balance in plants under Cd contamination. For instance, Zhang et al. [37] reported that biochar enhanced maize (*Zea mays* L.) growth by improving antioxidant defense mechanisms and reducing oxidative damage. Similarly, Liu et al. [38] found that biochar, when applied with metal-tolerant bacteria, alleviated Cd toxicity in rice by modulating antioxidant responses and improving soil properties.

Malondialdehyde (MDA) is a critical marker of lipid peroxidation and oxidative stress, while proline functions as an osmoprotectant under abiotic stress conditions. Our study showed that Cd_2_-treated plants had the highest MDA and proline levels, indicating severe oxidative damage. However, biochar, especially at 5%, significantly reduced MDA and proline accumulation, suggesting enhanced cellular protection against Cd toxicity. These findings are consistent with previous studies demonstrating the role of biochar in mitigating oxidative stress in Cd-exposed plants. For instance, Zhang et al. [37] reported that biochar application significantly lowered MDA levels in maize, reducing lipid peroxidation and oxidative damage. Similarly, Liu et al. [38] observed that biochar enhanced proline accumulation in rice (*Orzya sativa* L.), improving stress tolerance and osmotic balance under Cd stress.

One of the most concerning aspects of Cd toxicity is its high translocation from roots to shoots. Unlike other metals, Cd lacks effective exclusion mechanisms and is readily transported via the xylem, leading to high accumulation in leaves [39]. Our study confirmed that plants under Cd_2_ conditions had significantly higher Cd concentrations in leaves compared to Cd_1_. However, B-grown plants, particularly at 5%, exhibited reduced Cd accumulation, indicating lower Cd uptake from the soil. This aligns with previous findings demonstrating the role of biochar in limiting Cd bioavailability and translocation. For instance, Houben et al. [32] observed that biochar application decreased Cd uptake in rapeseed (*Brassica napus* L.) by enhancing metal immobilization in the rhizosphere. Moreover, Bandara et al. [40] highlighted that biochar amendments significantly restricted Cd mobility in lettuce, leading to lower shoot accumulation.

Our results confirm the effectiveness of biochar in reducing the bioavailability of Cd in soil, with significant effects on both the bioaccumulation factor (BAF) and the immobilization index (II). The decreasing trend of BAF with increasing biochar amendment suggests that Cd uptake by plants is reduced, likely due to its immobilization within the soil matrix. This effect is more pronounced at the lower Cd concentration (5 mg kg^−1^), where BAF decreases from 1.10 to 0.75 with 5% B. At higher concentrations (15 mg kg^−1^), a similar, but less marked, reduction is observed, indicating that biochar efficiency may depend on the saturation of adsorption sites. Similarly, the increase in II with rising biochar levels supports this hypothesis, showing higher values at 5 mg kg^−1^ compared to 15 mg kg^−1^, suggesting greater immobilization efficiency at lower Cd concentrations. Overall, these findings highlight the potential of biochar as a mitigation strategy for soils contaminated with Cd, although its effectiveness may vary depending on the initial metal concentration and the applied dose of biochar. Recent studies have substantiated the efficacy of biochar in reducing Cd bioavailability in soils, thereby decreasing its uptake by plants. For instance, Han et al. [41] demonstrated that biochar amendments decreased soil bioavailable Cd by up to 27.1% and reduced Cd accumulation in brown rice by up to 71.8%, leading to a significant increase in rice yield.

The European Union has established a maximum allowable concentration of 0.20 mg/kg fresh weight for Cd in leafy vegetables [42], which roughly corresponds to 4.0 mg/kg on a dry weight basis, considering a 95% water content (measured in our samples). In our study, lettuce plants grown with a soil Cd concentration of 5 mg/kg and without biochar exhibited a leaf Cd concentration of ca. 5.5 mg/kg dry weight, likely exceeding the legal limit on a fresh weight basis. A similar concentration was observed in plants treated with B_2_, which failed to reduce Cd accumulation significantly. However, the application of B_5_ resulted in a decrease to 3.5 mg/kg dry weight, likely bringing the Cd concentration below the legal threshold for fresh weight and ensuring compliance with food safety standards.

At Cd_2_, leaf Cd levels reached 13.5 mg/kg_DW_, with significant reductions in B_2_ (6.5 mg/kg dry weight) and B_5_ (6.0 mg/kg dry weight). However, despite these decreases, plants grown at Cd_2_ likely remained above the legal limit for fresh weight, indicating that in highly contaminated soils, biochar alone may not be sufficient to ensure that Cd concentrations fall within allowed levels. These results highlight the effectiveness of biochar, particularly at higher concentrations, in mitigating Cd uptake.

These findings are consistent with previous research demonstrating biochar’s ability to reduce Cd bioavailability in soils, thereby decreasing its translocation to edible plant tissues. Kim et al. [43] observed a similar trend in lettuce, where biochar amendments lowered Cd uptake by modifying soil chemical properties. Additionally, ur Rehman et al. [44] found that biochar significantly reduced Cd accumulation in wheat–rice systems irrigated with contaminated water. The observed reductions in our study suggest that biochar can be an effective strategy for mitigating Cd contamination in food crops [28].

### 3.2. Copper

Copper (Cu), for plants, is an essential micronutrient required for various physiological processes, including electron transport, enzymatic activity, and oxidative stress regulation. However, at high concentrations, Cu becomes toxic, leading to oxidative stress, chlorosis, and metabolic imbalances [45]. Unlike Cd, Cu uptake is tightly regulated, with plants restricting its translocation to prevent excessive accumulation in leaves [46]. Our study found that biochar effectively mitigated Cu toxicity, particularly at 5%, by reducing oxidative stress and enhancing plant resilience.

Excess Cu disrupts cell division, inhibits root elongation, and affects nutrient uptake, leading to reduced biomass accumulation [45]. Our results showed that Cu_2_-treated plants had significantly lower fresh weight compared to Cu_1_, confirming the dose-dependent toxicity of Cu. However, biochar amendment, particularly at 5%, improved fresh weight in Cu_2_-treated plants, suggesting enhanced growth despite Cu stress. These findings align with previous studies demonstrating that biochar can alleviate Cu toxicity; for instance, Buss et al. [47] found that applying 4% biochar to soil contaminated (200 mg/kg) with Cu improved the growth of quinoa (*Chenopodium quinoa* Willd.) to levels comparable to control plants without Cu stress. Similarly, Meier et al. [48] demonstrated that chicken-manure-derived biochar reduced Cu bioavailability in contaminated soils, leading to enhanced plant growth. Additionally, Wang et al. [49] showed that biochar amendments can increase soil pH, reduce exchangeable Cu, and enhance Cu binding to organic matter, thereby decreasing its phytotoxicity.

Cu toxicity interferes with chlorophyll synthesis by replacing essential cofactors like Mg^2+^ and Fe^2+^ in chlorophyll precursors, leading to chlorosis and impaired photosynthesis [50]. Our study revealed a significant reduction in chlorophyll content in Cu_2_-treated plants, with B_0_ plants showing the most severe decline. However, biochar application, particularly at B_5_, significantly restored chlorophyll levels, indicating reduced Cu uptake and enhanced photosynthetic stability. Similarly, Houben et al. [32] found that biochar amendment significantly increased the chlorophyll content in rapeseed grown in Cu-contaminated soil. Similarly, Zada et al. [51] observed that the application of biochar enhanced chlorophyll stability and photosynthetic activity in tomatoes (*Solanum lycopersicum* L.) under Cu stress.

A high soil Cu content induces ROS production, triggering antioxidant responses, including increased phenolic and flavonoid biosynthesis [52]. Our study found that Cu_2_ conditions significantly increased TPC and TFC levels in control plants, indicating an upregulation of secondary metabolites to counteract oxidative stress. However, plants grown with biochar exhibited a more balanced antioxidant response, with moderate TPC and TFC levels. These findings align with the results of Quartacci et al. [53] on lettuce grown in Cu-contaminated soil (100 mg/kg), where the plants grown with 5% biochar showed a reduction in TPC and TFC by ca. 30% and 25%, respectively, compared to untreated plants.

Similar to Cd, Cu toxicity induces oxidative stress, leading to increased MDA and proline levels. Our study showed that Cu_2_-treated plants had significantly higher MDA and proline content, confirming the damaging effects of Cu toxicity. However, biochar application significantly reduced both markers, particularly at 5%, suggesting a protective effect against Cu-induced oxidative damage. These findings are consistent with previous research demonstrating the role of biochar in mitigating oxidative stress caused by toxic metals. For instance, Naveed et al. [54] reported that biochar treatment decreased proline accumulation by 31% in rapeseed under Cu stress, suggesting improved osmotic regulation and stress tolerance.

Unlike Cd, which is freely translocated within plant tissues, Cu uptake and translocation are tightly regulated [55]. Copper is predominantly retained in root tissues and sequestered in vacuoles to prevent toxic accumulation in leaves [56]. Our study confirmed that despite high Cu exposure levels, Cu accumulation in leaves remained significantly lower than the soil concentration, indicating restricted translocation. Biochar further reduced Cu accumulation, particularly at 5%, suggesting that it effectively adsorbed Cu in the soil, preventing excessive uptake. These findings align with Buss et al. [47], where an amendment with 4% biochar to soil contaminated with Cu (200 mg/kg) significantly improved plant growth and reduced Cu concentrations (−43%) in quinoa. Similarly, Salmani et al. [57] showed that 2% B reduced growth in Cu-contaminated soil and application of 2% biochar significantly reduced Cu accumulation (−38%) in sunflower (*Helianthus annuus* L.) leaves.

Our findings indicate that the application of biochar has a limited effect on Cu uptake by plants, as reflected in the consistently low BAF values across all treatments. At Cu_1_, BAF remained stable at all biochar additions, while at Cu_2_ a slight decrease was observed, with BAF values reducing under B_2_ and B_5_. This suggests that Cu bioavailability in the soil is inherently low, likely due to strong adsorption to soil particles or plant-specific uptake mechanisms that regulate Cu accumulation. In contrast, the II for Cu exhibits a clear increasing trend with an increasing biochar application, particularly at Cu_2_. This indicates that biochar contributes to Cu immobilization within the soil matrix, though its impact on plant uptake is less pronounced than Cd. These findings align with Han et al. [41], who highlighted the role of biochar in toxic metal immobilization, but also suggested that its efficacy depends on the specific metal’s chemical properties and interactions with soil components. Specifically, it was shown that biochar amendment can significantly decrease the exchangeable fraction of Cu in the soil, effectively immobilizing the metal and reducing its mobility by increasing the soil pH and decreasing the exchangeable Cu fraction by 5 to 10 times, depending on the type of biochar used, leading to enhanced Cu immobilization [58].

While the European Union does not specify a maximum allowable Cu concentration for leafy vegetables [42], a reference value of 30 mg/kg_FW_, corresponding to 500 mg/kg_DW_ with a 95% moisture content, is often considered for risk assessment. In our study, lettuce plants grown in the soil Cu_1_ condition had leaf Cu concentrations of approximately 40 mg/kg_DW_ across all treatments, remaining well below the regulatory threshold. This suggests that Cu, unlike Cd, does not pose a significant risk for food safety in this context. In the Cu_2_ condition, leaf Cu levels reached 55 mg/kg_DW_ but were reduced to 35 mg/kg_DW_ in plants treated with both B_2_ and B_5_. Although these concentrations remain well below the safety threshold, the decrease suggests that biochar effectively limits Cu accumulation, potentially reducing its phytotoxic effects on plant metabolism. However, compared to Cd, the impact of biochar on Cu uptake appears less pronounced, likely due to the different soil interactions and plant regulatory mechanisms controlling Cu absorption. These results align with previous studies demonstrating the role of biochar in influencing Cu mobility in soils. Umaira Shams et al. [59] found that the application of biochar altered Cu availability in lettuce, affecting nutrient uptake and enzyme activity. While Cu concentrations in our study remained well below the safety limits regardless of treatment, the observed reductions with biochar suggest that its application may be beneficial in Cu-contaminated soils to enhance plant tolerance and overall health.

## 4. Materials and Methods

### 4.1. Experimental Setup

Before starting the experiment, soils were prepared by adding cadmium (Cd) and copper (Cu). In the laboratory, solutions were prepared for both metals at concentrations of 5 mg kg^−1^ and 15 mg kg^−1^ for Cd and 200 mg kg^−1^ and 600 mg kg^−1^ for Cu, using cadmium chloride (CdCl_2_) and copper chloride (CuCl_2_), respectively. These solutions were then used to artificially contaminate the universal potting soil (Vigor-Plant Italia srl, Fombio, Italy) serving as the substrate (Cd < 1 mg kg^−1^, Cu < 20 mg kg^−1^). The contaminated soil was subsequently oven-dried at 105 °C for three days. It is important to note that although the legal limit for these elements refer to their total concentrations, we have provided them in a readily bioavailable form.

**Figure 6:**
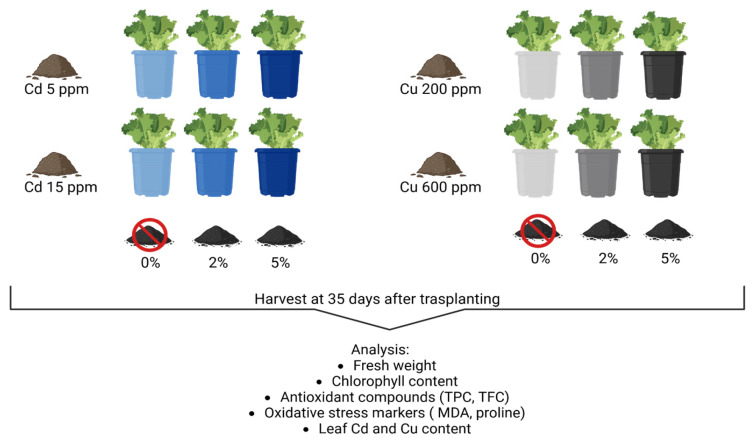
Experimental scheme.

For each concentration of the two metals, two biochar (B) amendments (*w*/*w*) were applied: 2% (B_2_) and 5% (B_5_); 0% (B_0_) was also used as control (Figure 6). The physicochemical characteristics of biochar are reported in Table 2.

At this stage, lettuce seedlings (*Lactuca sativa* L. cv Salanova), purchased from a local nursery (Vivaio Cerretani, Siena, Tuscany, Italy), were removed from their containers and transplanted (one seedling × pot) into black plastic pots (10 × 10 × 10 cm) containing the previously prepared soil. A total of 120 pots were prepared (60 for Cd and 60 for Cu), with 10 pots per treatment (statistical replicates). Plants were grown for five weeks in a climate-controlled chamber at 23 °C, 60% relative humidity, and 350 μmol m^−2^ s^−1^ PAR light intensity, with a 16/8 h day/night cycle. During the experimental period, plants were watered every three days with deionized water (dH_2_O), maintaining the soil field capacity at 70% in each pot. At the end of the five-week growth period, only the above-ground edible parts of lettuce plants were harvested, weighed for fresh leaf biomass, and/or dried using a dry machine or stored at −20 °C for subsequent biochemical analyses. Lettuce was chosen because it is a widely consumed leafy vegetable [60], often cultivated in contaminated agricultural soils, and is known for its high capacity to accumulate metals in edible tissues.

### 4.2. Chlorophyll Content

The chlorophyll content of lettuce leaves was measured using a portable, non-destructive chlorophyll content meter (CCM-300, Opti-Sciences Inc., Hudson, NH, USA). For each plant, measurements were taken from three fully expanded upper leaves. Specifically, three measurements per leaf were recorded at distinct positions: near the tip, at the center, and close to the base, following the methodology outlined by Fedeli et al. [61]. The obtained values are expressed on a surface area basis as milligrams of chlorophyll per square meter of leaf (mg m^−2^).

### 4.3. Antioxidant Compounds

The total phenolic content (TPC) and total flavonoid content (TFC) were assessed following a modified version of the method described by Wakeel et al. [62]. Approximately 1 g of dried material was soaked in 10 mL of 80% methanol (*v*/*v*). The mixture was subjected to orbital shaking for 30 min and then incubated at 4 °C in the dark. After 48 h, the samples were filtered using Whatman filter paper no. 1, and the resulting filtrates were used for the determination of TPC and TFC.

#### 4.3.1. Total Phenolic Content

TPC determination was carried out according to the method outlined by Fedeli et al. [63]. Specifically, 0.125 mL of filtrate was combined with 2 mL of dH_2_O, followed by the addition of 0.125 mL of Folin–Ciocalteu’s reagent. After a 3 min incubation in the dark, 1.25 mL of 7% Na_2_CO_3_ and 1 mL of dH_2_O were added, and the mixture was shaken vigorously. The samples were then incubated in the dark for 90 min before measuring the absorbance at 760 nm using a UV–Vis spectrophotometer (Agilent 8453, Technologies, Santa Clara, CA, USA). A calibration curve (5–300 μg mL^−1^) was prepared using gallic acid as reference standard.

#### 4.3.2. Total Flavonoid Content

TFC determination was carried out according to the method outlined by Fedeli et al. [64]. A volume of 0.250 mL of filtrate was mixed with 0.075 mL of 5% NaNO_2_. After 5 min, 0.075 mL of 10% AlCl_3_ were added. Following another 5 min incubation in the dark, 0.5 mL of 1 M NaOH solution was added. The samples were further incubated in the dark for 15 min before measuring the absorbance at 415 nm using a UV–Vis spectrophotometer (Agilent 8453, Technologies, Santa Clara, CA, USA). For the quantification, a calibration curve (12.5–150 μg mL^−1^) was prepared using quercetin as reference standard.

### 4.4. Oxidative Stress Markers

#### 4.4.1. Malondialdehyde

The malondialdehyde (MDA) content was determined according to the method described by Azarnejad et al. [65]. Specifically, 0.5 g of the frozen sample was homogenized in 5 mL of an extraction solution containing 0.25% TBA dissolved in 10% TCA. The resulting mixture was incubated at 95 °C for 30 min in a thermoblock and then rapidly cooled on ice to halt the reaction. Following centrifugation at 4000 rpm for 10 min, the supernatant was collected, and its absorbance was measured at 532 nm and 600 nm using a UV–Vis spectrophotometer (Agilent 8453,Technologies, Santa Clara, CA, USA). The MDA concentration was calculated by subtracting the absorbance at 600 nm and applying an extinction coefficient of 155 mM^−1^ cm^−1^ for the MDA-TBA complex.

#### 4.4.2. Proline

The proline content was determined according to the method described by Fedeli et al. [66]. Specifically, 0.1 g of the frozen sample was homogenized with 2 mL of 3% 5-sulfosalicylic acid dihydrate and centrifuged at 4000 rpm for 10 min. Subsequently, 0.5 mL of the supernatant was mixed with 0.5 mL of glacial acetic acid and 0.5 mL of acid-ninhydrin reagent, which was prepared by dissolving 1.25 g of ninhydrin in 30 mL of glacial acetic acid and 20 mL of 6 M phosphoric acid. The mixture was then incubated at 100 °C for 1 h before being cooled on ice to stop the reaction. Finally, 1.5 mL of toluene was added, and the absorbance of the clear supernatant was measured at 520 nm using a UV–Vis spectrophotometer (Agilent 8453, Technologies, Santa Clara, CA, USA) A calibration curve was generated using a stock solution of 1 mM L-proline with concentrations ranging from 2 to 600 μL.

### 4.5. Cd and Cu Determination

#### 4.5.1. Cadmium

Dry samples (ca. 0.250 g) were subjected to acid digestion in a microwave-assisted system (Milestone Ethos 900, Bergamo, Italy) using 3 mL of 70% HNO_3_ and 0.5 mL of 30% H_2_O_2_ at a temperature of 280 °C and a pressure of 55 bar [67]. The concentration of Cd was determined by Inductively Coupled Plasma-Mass Spectrometry (ICP-MS) (Perkin Elmer NexION 350, MA, USA). Analytical quality was verified with the certified reference material NCS DC 73350 (‘*Poplar leaves*’), which yielded recovery rates in the range of 91–108%. The precision of the analysis, assessed through the coefficient of variation across five replicates, consistently exceeded 98%. The results are expressed on a dry weight basis (mg/kg).

#### 4.5.2. Copper

Dry samples (ca. 1 g) were directly analyzed using a portable X-ray fluorescence (XRF) device. The samples were placed in a plastic cup, which was then inserted into the designated compartment of the instrument. The content of Cu was measured in *Soil* mode, with the acquisition time set at 20 s per beam, for a total of three beams per analysis [68]. The accuracy of the analysis was validated using 14 certified plant matrices, as reported by Fedeli et al. [69]. The results are expressed on a dry weight basis (mg/kg).

### 4.6. Bioaccumulation Factor and Immobilization Index

To evaluate the accumulation and immobilization efficiency of Cd and Cu in plant tissues under different biochar treatments, two indices were calculated: the bioaccumulation factor (BAF) and the immobilization index (%) (II).

The BAF was determined to assess metal uptake and translocation from the soil to the aerial parts of the plant, and was calculated using the following equation [70]:

(1) $$BAF=\;\frac{Leaf\;\left[M\right]}{Soil\;\left[M\right]},$$

where

- *Leaf* [*M*] = content (mg/kg) of Cd or Cu in lettuce leaves;
- *Soil* [*M*] = initial concentration (mg/kg) of Cd or Cu in the soil.

The II was used to evaluate the biochar’s ability to reduce the bioavailability of metals in plants, and was calculated using the following equation [71]:

(2) $$II\;\left(\%\right)=\left(\frac{{\left[M\right]}_{Control}-{\left[M\right]}_{Sample}}{{\left[M\right]}_{Control}}\right)\times \;100,$$

where

- [*M*]*_Control_* = content (mg/kg) of Cd or Cu in leaves from lettuce plants grown in unamended soil (B_0_);
- [*M*]*_Sample_* = content (mg/kg) of Cd or Cu in leaves from lettuce plants grown in biochar-amended soils (B_2_ and B_5_).

### 4.7. Statistical Analysis

The data approached a normal distribution (Shapiro–Wilk test *p* > 0.05) and the results are thus presented as means ± standard error. To evaluate the effect of biochar and the two metals (Cd and Cu), a permutational analysis of variance (PERMANOVA) was run. When the main test yielded a *p*-value <0.05, indicating significant differences between treatments, a pairwise permutation *t*-test was run as a post hoc analysis (*p* < 0.05). All statistical analyses were performed using the R software (v.4.4.1) [72].

## 5. Conclusions

This study demonstrates the potential of biochar as a soil amendment to mitigate toxic metal stress in plants, with differing effects on Cd and Cu. Biochar effectively reduced Cd bioavailability, leading to improved plant growth and physiological responses, while its impact on Cu was primarily associated with immobilization in the soil rather than reduced plant uptake.

The results highlight the role of biochar in enhancing plant resilience to metal stress by improving soil conditions and reducing oxidative damage. While Cd accumulation was significantly reduced with biochar application, Cu uptake remained low across all treatments, suggesting that Cu bioavailability is naturally restricted. The increasing immobilization index along with higher biochar levels further confirm its role in stabilizing both metals in the soil. Overall, these findings support the use of biochar as a sustainable strategy for soil remediation, particularly in Cd-contaminated environments.

## Figures and Tables

**Table 1:** Bioaccumulation factor (BAF) and immobilization index (II) (mean ± SE, *n* = 10) of lettuce plants grown in soil contaminated with Cd and Cu. Cd_1_ = Cd 5 mg kg^−1^; Cd_2_ = Cd 15 mg kg^−1^; Cu_1_ = Cu 200 mg kg^−1^; Cu_2_ = Cu 600 mg kg^−1^. B_0_ = biochar 0%; B_2_ = biochar 2%; B_5_ = biochar 5%. Different letters indicate statistically significant differences between treatments (*p* < 0.05).

**BAF**				
	*Cd*_1_	*Cd*_2_	*Cu*_1_	*Cu*_2_
*B*_0_	1.10 ± 0.10 ^a^	0.76 ± 0.07 ^b^	0.03 ± 0.01 ^a^	0.008 ± 0.001 ^b^
*B*_2_	1.04 ± 0.08 ^a^	0.54 ± 0.05 ^c^	0.03 ± 0.01 ^a^	0.005 ± 0.001 ^c^
*B*_5_	0.75 ± 0.06 ^b^	0.56 ± 0.05 ^c^	0.03 ± 0.01 ^a^	0.005 ± 0.001 ^c^
**II (%)**				
	*Cd*_1_	*Cd*_2_	*Cu*_1_	*Cu*_2_
*B*_0_	-	-	-	-
*B*_2_	5.46	29.80	2.98	27.1
*B*_5_	31.89	27.02	1.85	34.1

**Table 2:** Physiochemical characteristics of biochar.

Particle diameter (µm)	<200
Total N (%)	<0.4
Total K (mg kg^−1^)	3020
Total P (mg kg^−1^)	340
Total Ca (mg kg^−1^)	9920
Total Mg (mg kg^−1^)	852
Total Na (mg kg^−1^)	291
C from carbonate (%)	<0.1
Total Cd (mg kg^−1^)	<1
Total Cu (mg kg^−1^)	30
Total C (%)	68.7
WHC (%)	23.5
Salinity (mS m^−1^)	110
pH	9.9
Hash content (%)	4.6
H/C	0.2

## Data Availability

Data are available on reasonable request from the corresponding author.
